# Grand multiparity as a predictor of adverse pregnancy outcome among women who delivered at a tertiary hospital in Northern Tanzania

**DOI:** 10.1186/s12884-019-2377-5

**Published:** 2019-07-02

**Authors:** Zainab Muniro, Clifford Silver Tarimo, Michael J. Mahande, Eusebious Maro, Bariki Mchome

**Affiliations:** 1 Department of Obstetrics and Gynecology, Kilimanjaro Christian Medical Centre, Box 3010, Moshi, Tanzania; 2 Department of Epidemiology and Biostatistics, Institute of Public Health, Kilimanjaro Christian Medical University College, Box 2240, Moshi, Tanzania; 3 Dar es Salaam Institute of Technology, Department of Science and Laboratory Technology, Box 2958, Dar es Salaam, Tanzania

**Keywords:** Prevalence, Grand multiparity, Low parity, Pregnancy outcomes, North-Tanzania

## Abstract

**Background:**

Grand multiparity has been associated with increased risks of adverse pregnancy outcomes such as post-partum hemorrhage,gestational hypertension, gestationaldiabetes mellitus and high perinatal mortality.There is limited information about the impact of high parity on pregnancy outcomes in Tanzania. This study aimed to determine prevalence, trend and associated adverse pregnancy outcomes of grand multiparity in a tertiary hospital in Northern Tanzania.

**Methods:**

A retrospective cross-sectional study was conducted at Kilimanjaro Christian Medical Centre (KCMC) using maternally linked data from medical birth registry. Women with singleton deliveries from 2006 to 2014 were analyzed. The prevalence of grand-multiparity was computed as proportion to estimate the trend over years. Adverse pregnancy outcomes associated with grand multiparity were estimated using multivariable logistic regression models. A *p*-value of < 0.05 was considered statistically significant.

**Results:**

The overall prevalence of grand multiparity was 9.44% ranging from 9.72% in 2006 to 8.49% in 2014. The grand multiparous women had increased odds of prelabour rupture of membranes (Adjusted odds ratio [AOR] 1.78: 95% CI:1.28–2.49), stillbirth (AOR 1.66: 95% CI:1.31–2.11) and preterm birth delivery (AOR 1.28; 95% CI: 1.05–1.56) as compared to women in the lower parity group.

**Conclusions:**

The prevalence of grand multiparity among women in North-Tanzania was 9.44%. It was significantly associated with adverse pregnancy outcomes. This calls for a need to increase community awareness on its risks, encourage birth control among older women. Delivery-care facilities should prepare for emergency situation when attending deliveries of high parity group.

## Background

As Sustainable Development Goal’s (SDG) efforts to address promotion of maternal health and reduction of child mortality becomes intensified, regional specific investigation to inform the public health on potential effect of factors that presents a significant risk to the pregnant mother and the birth outcome are imperative. Huge disparity in the fertility rate exist between the developed and developing countries. Low-and-middle income countries including Tanzania’s fertility rate have still relatively high annual fertility rate as compared to high income countries [1]. This trend may pose substantial risk to the pregnant mother and result in adverse birth outcome. Among other factors, grand multiparity is hypothesized to play a significant role in adverse maternal outcomes especially in low-and-middle income countries like Tanzania.

Grand-multiparity (GMP) condition has been defined differently in several literature [1–3]. Some literature define it as a woman who delivered four or more times while others consider of six or more deliveries [4]. Furthermore, the International Federation of Gynecology and Obstetrics defined GMP as deliveries of a fifth to ninth, while naming those undergoing their tenth or more deliveries as great grand-multiparous or huge great grand-multiparous [5–8]. According to the CDC report of 2004, the prevalence of grand multiparity was reported as low as 3–4% in developed countries as compared to 19.3% in developing countries, [9, 10]. The lower prevalence in developed countries has been attributed to high use of modern family planning and optimal obstetric care while the high prevalence of GMP in low income countries is fueled by gender desirability, low education and desire for more offspring to have large family size [11, 12]. Adverse outcomes associated with GMP include diabetes, premature labor, maternal and perinatal mortality, placenta previa, genital sepsis, postpartum hemorrhage (PPH), utero-vaginal prolapse, hypertension and Intrauterine fetal death (IUFD) [6, 7, 10, 13]. Controversy prevails in the effect of high parity on these complications since some other studies report no increased incidences of obstetric complications [1, 14]. Despite efforts to reduce maternal and perinatal adverse outcomes in Tanzania, maternal morbidities and mortalities are high. This study takes advantage of existing large scale birth registry data to reaffirm existing small scale evidence conducted in 2013 in one of the tertiary facility in Tanzania and attempt to account for change in magnitude of grandmultiparity overtime with an additional capability to capture infrequent adverse outcome. Moreover, describing a trend of grand multiparity overtime may provide a reflection of effectiveness of contraceptive coverage in this population. This study’s primary aim was to determine the association between GMP and adverse pregnancy outcome. Moreover, we aimed at assessing the prevalence, trend and associated pregnancy outcomes of grand multiparity in a tertiary hospital in Northern Tanzania over nine (9) years.

## Methods

### Study design, setting & population

**Fig. 1:**
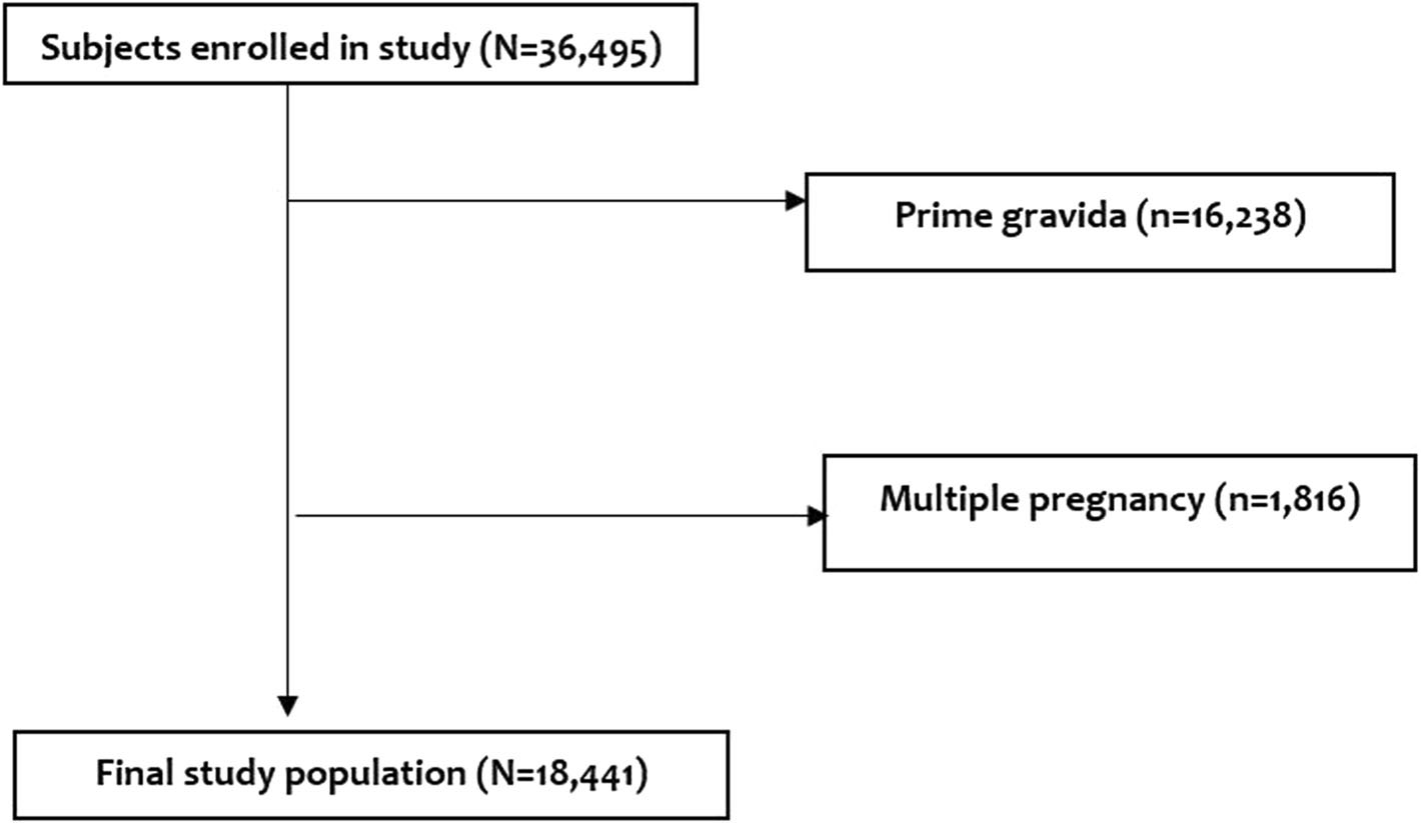
Schematic Diagram of the selected study participants

This was a cross-sectional study using maternally linked data from Kilimanjaro Christian Medical Centre (KCMC) medical birth registry. KCMC is a referral consultants and teaching hospital located in Moshi, Kilimanjaro region in Northern-Tanzania. According to the Tanzania National Census of 2012, the population of Kilimanjaro region is estimated to be 1.6 million people. The average deliveries per year is around 4000. The KCMC medical birth registry was established in the year 1999 as pilot and officially started to operate in 2000. Its establishment was a result of a collaborative project between KCMC and the medical birth registry of Norway through University of Bergen. Since then, medical records for all women who deliver at KCMC and their newborns have been prospectively collected and stored at the medical birth registry. In this study, multiparous women (parity of 2 or more) of 28 weeks of gestation or more, with singleton births from the year 2006 to 2014 were studied. We excluded multiple pregnancy to avoid overrepresentation of high-risk women which might exaggerate the pregnancy outcomes. The final sample analyzed comprised of 18,441 deliveries (Fig. 1).

### Data collection methods and tools

Socio-demographic and obstetric information for all women who delivered at KCMC were recorded at the medical birth registry in an electronic database. Information for all deliveries that occur at the department of Obstetrics and Gynecology are prospectively collected and entered in a computerized database system at the medical birth registry. Trained midwife nurses conduct interviews on daily basis using a standardized questionnaire for all women who deliver at the hospital within 24 h of delivery or immediately after recovery from a complicated delivery. Information on the neonates who are admitted in the neonatal care unit were recorded in neonatal registry forms. Apart from birth registry, each pregnant woman attending this facility has her history documented on patient file with unique file number. These files are confidential and contain all patients’ medical records stored at the hospital medical record department. Other patients’ information is entered and kept in various registers at each service delivery points such as theater rooms, labor ward, Intensive Care Unit (ICU) and in the antenatal and postnatal units. Data extraction sheet was used to obtain information from demographic characteristics and immediate maternal and fetal complications.

### Definition of variables

The main outcome variables include prelabour rupture of membranes (PROM), postpartum hemorrhage (PPH), pre-eclampsia, low birth weight, stillbirth and preterm birth. Still birth was defined as fetal death of ≥28 weeks of gestation age while preterm birth was defined as babies born alivebefore 37 weeks of pregnancy are completed. Low birth weight (LBW) was defined as a birth weight of less than 2500 g with gestational age of ≥28 weeks. Independent variables included parity status as the main exposure whereby multiparity was defined as a parity of 2 to 4 deliveries while grand multiparity was defined as a parity of 5 or more deliveries. Maternal socio-demographic characteristics included maternal age, residence, level of education, religion, number of antenatal care ANC-visits, gestational age, body mass index (BMI) and marital status.

### Data analysis

Data analysis was performed using STATA 12.0 (StataCorp LP, College Station, Texas 77,845 USA). After data extraction from the main database, data were cleaned, checked for consistency and verification of missing values. Descriptive statistics were summarized using frequency and percentages for categorical variables. Trend in grand-multiparity was determined by calculating yearly proportions and displayed using a line graph. Chi-square test was used to determine the association between a set of maternal pregnancy outcomes and GMP. Odds ratio (OR) and 95% confidence interval (CI) for adverse maternal and fetal outcomes associated with GMP were estimated using multivariable logistic regression models. A *p*-value of less than 0.05 (2-tailed) was considered statistically significant.

## Results

### Demographic and obstetrics characteristics of the study population

A total of 18,441 deliveries were analyzed. Prevalence of grand multiparity was 9.44%. The chi-square test for linear trend showed that, there appears to be a statistically significant trend in prevalence of grand multiparity over years (*p* < 0.001). Demographic and obstetrics characteristics of the study participants are summarized in Table 1. The mean age of women in the study group was 30.4 (SD = 5.6) years. About 64% of grand multiparous women in this study were aged > 35 years and more than half (62.5%) resided in rural area of North-Tanzania. Approximately half (52%) of the grand multiparous subjects had less than 4 antenatal care visits during their pregnancy while 60% delivered at term.

**Table 1:** Distribution of pregnancy outcome by parity and selected characteristics

Characteristic	Pregnancy outcome											
	Pre-eclampsia		PROM		PPH		Still birth		LBW		Preterm	
	Yes n (%)	No n (%)	Yes n (%)	No n (%)	Yes n (%)	No n (%)	Yes n (%)	No n (%)	Yes n (%)	No n (%)	Yes n (%)	No n (%)
Parity												
Multipara	595 (86.36)	16,106 (90.73)	239 (83.57)	16,462 (90.67)	319 (85.07)	16,382 (90.68)	547 (80.8)	16,154 (90.94)	1704 (87.65)	14,997 (90.91)	2023 (87.69)	14,678 (90.98)
GMP	94 (13.64)	1646 (9.27)	47 (16.43)	1693 (9.33)	56 (14.93)	1684 (9.32)	130 (19.2)	1610 (9.06)	240 (12.35)	1500 (9.09)	284 (12.31)	1456 (9.02)
Maternal age												
< 25	64 (9.29)	2700 (15.21)	40 (13.99)	2724 (15.0)	39 (10.4)	2725 (15.08)	86 (12.7)	2678 (15.08)	312 (16.05)	2452 (14.86)	373 (16.17)	2391 (14.82)
25–29	161 (23.37)	5324 (29.99)	77 (26.92)	5408 (29.79)	117 (31.2)	5368 (29.71)	178 (26.29)	5307 (29.88)	546 (28.09)	4939 (29.94)	658 (28.52)	4827 (29.92)
30–34	225 (32.66)	5597 (31.53)	90 (31.47)	5732 (31.57)	105 (28.0)	5717 (31.65)	202 (29.84)	5620 (31.64)	583 (29.99)	5239 (31.76)	689 (29.87)	5133 (31.81)
> 35	239 (34.69)	4122 (23.22)	79 (27.62)	4282 (23.59)	113 (30.13)	4248 (23.51)	210 (31.02)	4151 (23.37)	501 (25.77)	3860 (23.4)	583 (25.27)	3778 (23.42)
Missing	0 (0.0)	9 (0.05)	0 (0.0)	9 (0.05)	1 (0.27)	8 (0.04)	1 (0.15)	8(0.05)	2 (0.1)	7 (0.04)	4 (0.17)	5 (0.03)
Maternal BMI												
Underweight	11 (1.60)	426 (2.4)	4 (1.4)	433 (2.39)	6 (1.6)	431 (2.39)	12 (1.77)	425 (2.39)	67 (3.45)	370 (2.24)	61 (2.64)	376 (2.33)
Normal weight	121 (17.56)	5229 (29.46)	63 (22.03)	5287 (29.12)	97 (25.87)	5253 (29.08)	138 (20.38)	5212 (29.34)	510 (26.23)	4840 (29.34)	620 (26.87)	4730 (29.32)
Overweight	134 (19.45)	3487 (19.64)	51 (17.83)	3570 (19.66)	58 (15.47)	3563 (19.72)	95 (14.03)	3526 (19.85)	303 (15.59)	3318 (20.11)	353 (15.30)	3268 (20.26)
Obese	153 (22.21)	1962 (11.05)	35 (12.24)	2080 (11.46)	32 (8.53)	2083 (11.53)	46 (6.79)	2069 (11.65)	151 (7.77)	1964 (11.91)	209 (9.06)	1906 (11.81)
Missing	270 (39.19)	6648 (37.45)	133 (46.50)	6785 (37.37)	182 (48.53)	6736 (37.29)	386 (57.02)	6532 (36.77)	913 (46.97)	6005 (36.40)	1064 (46.12)	5854 (36.28)
ANC visits												
< 4	312 (45.28)	7094 (39.96)	131 (45.8)	7275 (40.07)	163 (43.47)	7243 (40.09)	381 (56.28)	7025 (39.55)	1132 (58.23)	6274 (38.03)	1441 (62.46)	5965 (36.97)
4+	367 (53.27)	10,459 (58.92)	152 (53.15)	10,674 (58.79)	197 (52.53)	10,629 (58.83)	273 (40.32)	10,553 (59.41)	753 (38.73)	10,073 (61.06)	816 (35.37)	10,010 (62.04)
Missing	10 (1.45)	199 (1.12)	3 (1.05)	206 (1.13)	15 (4.0)	194 (1.07)	23 (3.4)	186 (1.05)	59 (3.03)	150 (0.91)	50 (2.17)	159 (0.99)
Education level												
None	7 (1.02)	372 (2.10)	4 (1.4)	375 (2.07)	13 (3.47)	366 (2.03)	28 (4.14)	351 (1.98)	59 (3.03)	320 (1.94)	38 (1.65)	341 (2.11)
Primary	388 (56.31)	10,799 (60.83)	190 (66.43)	10,997 (60.57)	243 (64.8)	10,944 (60.58)	490 (72.38)	10,697 (60.22)	1283 (66.0)	9904 (60.04)	1528 (66.23)	9659 (59.87)
Secondary	93 (13.50)	1938 (10.92)	34 (11.89)	1997 (11.0)	32 (8.53)	1999 (11.06)	51 (7.53)	1980 (11.15)	211 (10.85)	1820 (11.03)	263 (11.4)	1768 (10.96)
Tertiary	201 (29.17)	4617 (26.01)	58 (20.28)	4760 (26.22)	83 (22.13)	4735 (26.21)	104 (15.36)	4714 (26.54)	386 (19.86)	4432 (26.87)	470 (20.37)	4348 (26.95)
Missing	0 (0.0)	26 (0.15)	0 (0.0)	26 (0.14)	4 (1.07)	22 (0.12)	4 (0.59)	22 (0.12)	5 (0.26)	21 (0.13)	8 (0.35)	18 (0.11)

### Trend of grand multiparity

**Fig. 2:**
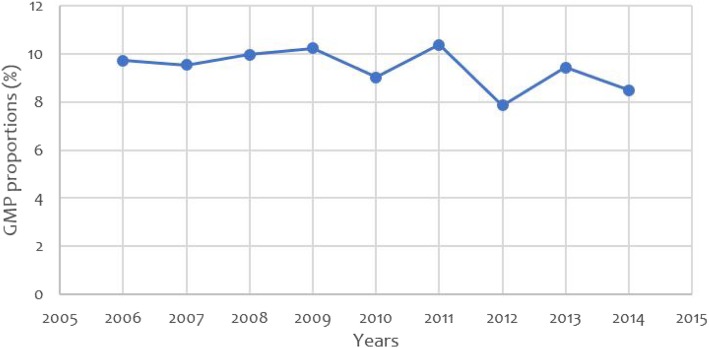
Trend of grand multiparity from 2006 to2014 at KCMC

Trend of GMP among women who delivered at KCMC from 2006 to 2014 was seen to decrease over years. Although there is variation in each year, the proportion in the year 2006 (9.72%) and 2007 (9.03%) are rather similar. The highest proportion was appreciated in the year 2011 (10.38%) followed by 7.85, 9.43 and 8.49% in the years 2012, 2013 and 2014 respectively (Fig. 2).

### Association between pregnancy outcomes and grand multiparity

The Chi-square test for pregnancy outcomes and parity is shown in Table 2. In this test, all pregnancy outcomes assessed in the study were significantly associated with parity status of participants.

**Table 2:** Association between parity and pregnancy outcomes

Parity	Pregnancy outcome																	
	Pre-eclampsia			PROM			PPH			Still birth			LBW			Preterm		
	Yes n (%)	No n (%)	*p*-value	Yes n (%)	No n (%)	*p*-value	Yes n (%)	No n (%)	*p*-value	Yes n (%)	No n (%)	*p*-value	Yes n (%)	No n (%)	*p*-value	Yes n (%)	No n (%)	*p*-value
Multipara	595 (86.36)	16,106 (90.73)	< 0.001	239 (83.57)	16,462 (90.67)	< 0.001	319 (85.07)	16,382 (90.68)	< 0.001	547 (80.8)	16,154 (90.94)	< 0.001	1704 (87.65)	14,997 (90.91)	< 0.001	2023 (87.69)	14,678 (90.98)	< 0.001
GMP	94 (13.64)	1646 (9.27)		47 (16.43)	1693 (9.33)		56 (14.93)	1684 (9.32)		130 (19.2)	1610 (9.06)		240 (12.35)	1500 (9.09)		284 (12.31)	1456 (9.02)	

The crude and adjusted analysis for the association between grand multiparity and adverse pregnancy outcomes are displayed in Table 3 and Table 4 respectively. After adjusting for maternal age, maternal education, mothers’ residence, body mass index (BMI), number of antenatal care visits and gestational age, PROM (AOR 1.78; 95% CI: 1.28–2.49) remained significantly associated with grand multiparity. When fetal outcomes were adjusted for the same factors, stillbirth (AOR 1.66; 95% CI: 1.31–2.11) and preterm birth (AOR 1.28; 95% CI: 1.05–1.56) remained statistically significant factors associated with GMP (Table 4).

**Table 3:** Model 1: Unadjusted effects of parity on pregnancy outcome

Parity	Pregnancy outcome											
	Pre-eclampsia		PROM		PPH		Still birth		LBW		Preterm	
	COR	95% CI	COR	95% CI	COR	95% CI	COR	95% CI	COR	95% CI	COR	95% CI
Multipara^RC^	1		1		1		1		1		1	
GMP	1.55	1.24–1.93	1.91	1.39–2.62	1.71	1.28–2.28	2.38	1.95–2.91	1.41	1.22–1.63	1.42	1.24–1.62

Note: RC (Reference category)

**Table 4:** Model 2: Adjusted effect of parity on pregnancy outcome

Parity	Pregnancy outcome											
	Pre-eclampsia		PROM		PPH		Still birth		LBW		Preterm	
	AOR	95% CI	AOR	95% CI	AOR	95% CI	AOR	95% CI	AOR	95% CI	AOR	95% CI
Multipara^RC^	1		1		1		1		1		1	
GMP	1.32	0.94–1.85	1.78	1.28–2.49	1.34	0.95–1.9	1.66	1.31–2.11	1.26	0.97–1.62	1.28	1.05–1.56

Note: RC (Reference category)

## Discussion

The prevalence of grand multiparity at the institution over nine years found was found to be 9.44%. This study found that grand multiparity was associated with maternal and perinatal complications such as prelabour rupture of membranes, stillbirth and preterm birth. The prevalence of grand multiparity in this study was consistent with a study done in Saudia by Alsammamiet al which reported a prevalence of 5.3% [15]. However, this is slightly lower compared to other studies which reported the high prevalence of 10.2 and 26.5% respectively [9, 13]. The higher prevalence in the later studies could be explained by high prevalence of young marriage and poor acceptance of modern family planning methods. The lower prevalence in this study can be attributed to standard literacy level of grand multiparas, as over 52% had at least primary level of education.

In the present study, we found that the trend of grand multiparity to be decreasing over nine years.. This decline could have a broader range of attributes including availability of higher education to women and increased community awareness on the health risks of giving birth at an advanced maternal age. The study site has better schools and universities that provides with an opportunity to learn and become aware of reproductive health status. However, the first five (5) years in the trend show no bigger differences while in the year 2011 the rise is seen and then followed by the decline. This can be explained by the fact that at this particular time, the country had adapted well to the strategic plan developed in 2008 known as One Plan which aimed to provide guidance and improve reproductive, maternal, newborn and child health [16].

In this study we found a significant increased risk of prelabour rupture of membranes (PROM) in grand multiparous women compared to those with low parity. Our result is consistent with previous studies done in Nigeria that describe a 3.6–4.2% increase in PROM in grand multiparas as compared to multiparas [16, 17]. Advanced maternal age (AMA) has been reported as a major risk factor in developing other maternal conditions including hypertension, diabetes mellitus, renal diseases, and other chronic infections which in turn are associated with PROM [18, 19]. This could be the case in the present study.

In multivariable regression analyses, our study did not find a significant association between PPH and GMP. This is in consistent with study done by Selo-Ojeme*et al* among Nigerian women [21]. Similar findings were reported by Combs *et al* who studied women from the United States [22]. We also found that GMP was significantly associated with an increased risk of PPH when controlled by maternal age and educational level attainment. However, addition of other covariates in the model led to non-significant association. Hence, it may suffice to say that, in this population, PPH is well explained by GMP condition, maternal age and education level. Due to increased maternal age and repeated deliveries, the uterine wall becomes weak resulting in inability to adequately contract leading to development of PPH [22]. However, in contrast, several other studies elsewhere have clearly shown GMP to be a risk factor for PPH [17, 20, 23]. Few data that didn’t demonstrate an increased risk for PPH in grand multiparity could be attributed to a small scale design with inability to capture rare adverse events [13].

This study has demonstrated that GMP women had an increased odds of experiencing stillbirth and preterm births as compared to women with low parity as previously described in published data from Uganda and Nigeria [9, 21, 22]. This could be explained by the fact that since AMA is a major risk for various maternal complications, thus fetus and neonates may always be susceptible to mortality and morbidity. Other fetal complications which have been reported in other studies in Tanzania with inadequately controlled intrapartum confounders include meconium stained liquor and low apgar score which were found to be insignificant complications in the present study.

### Strengths and limitations

This hospital-based study used a birth registry data collected routinely at KCMC using a standardized questionnaire. From this data set a large sample size was obtained thus giving the study more power and making it possible to adjust for confounders as well as making inference in similar settings with high precision. Despite the strengths, there were some limitations that should be considered while interpreting findings from this study. Since this was a referral hospital-based study, our results may not be generalizable to the general population of Tanzania. In attempt to minimize overrepresentation of high–risk women, we excluded women with multiple pregnancy. In addition, maternal mortality, as one of the important outcomes was not investigated in this study due to insufficient information regarding maternal deaths in the dataset. We therefore recommend future prospective study to explore the association of GMP with maternal mortality.

## Conclusion

Despite low prevalence of GMP at the institution, it continues to pose additional challenges to fetal and maternal health. Although the GMP proportion has decreased over years, our result reveals a rather gradual decline of GMP overtime. The study warrants more emphasis to be directed towards community sensitization on the adverse maternal and fetal outcomes that might be attributable to GMP. The study also suggests a need for establishment of a referral system unit at this institution that will be specifically managing deliveries occurring in women with high parity as well as advanced age.

## Data Availability

The datasets during the current study are not publicly available to protect the participants’ anonymity. But it can be freely available from the corresponding author on reasonable request.

## Abbreviations

AMA: Advanced maternal age
ANC: Antenatal clinic
AOR: Adjusted odds ratio
BMI: Body mass index
CI: Confidence interval
COR: Crude odds ratio
GMP: Grand multiparity
ICU: Intensive care unit
KCMC: Kilimanjaro Christian Medical Centre
LBW: Low birth weight
PROM: Prelabour rupture of membranes

## References

[1] Bugg GJ, Atwal GS, Maresh M (2002). Grand multiparae in a modern setting. BJOG An Int J Obstet Gynaecol.

[2] Opara EI, Zaidi J (2007). The interpretation and clinical application of the word “parity”: a survey. BJOG An Int J Obstet Gynaecol..

[3] Babinszki A, Kerenyi T, Torok O, Grazi V, Lapinski RH, Berkowitz RL (1999). Perinatal outcome in grand and great-grand multiparity: effects of parity on obstetric risk factors. Am J Obstet Gynecol.

[4] Chandra A, Copen CE, Stephen EH. Infertility and impaired fecundity in the United States, 1982-2010: data from the National Survey of family growth. Natl Health Stat Report. 2013;(67):1–18. 24988820

[5] Njoku CO, Abeshi SE, Emechebe CI (2017). Grand Multiparity : obstetric outcome in comparison with multiparous women in a developing country. Open J Obstet Gynecol.

[6] Eze JN, Okaro JM, Okafor MH. Outcome of pregnancy in the grand multipara in Enugu, Nigeria. Trop J Obstet Gynaecol. 2006;23:8–11 Available from: 10.4314/tjog.v23i1.14555.

[7] Kuti O, Dare F, Ogunniyi S (2001). Grand multiparity: mothers’ own reasons for the index pregnancy. Trop J Obstet Gynaecol.

[8] Solomons B (1934). The dangerous multipara. Lancet..

[9] Hoque M, Hoque E, Kader SB (2008). Pregnancy complications of grand multiparity at a rural setting of South Africa. Iran J Reprod Med.

[10] Mutihir J (2005). Obstetric outcomes of the grand multipara in Jos, Nigeria. J Med Trop.

[11] Maro Eusebious W., Mosha Neema R., Mahande Michael Johnson, Obure Joseph, Masenga Gileard (2016). Ten years trend in maternal mortality at Kilimanjaro Christian Medical Center Tanzania, 2003–2012: A descriptive retrospective tertiary hospital based study. Asian Pacific Journal of Reproduction.

[12] Emechebe C, Njoku C, Eyong E, Maduekwe K, Ukaga J (2016). The social class and reasons for grand multiparity in Calabar, Nigeria. Trop J Obstet Gynaecol.

[13] Ogbe AE, Ogbe BP, Ekwempu C (2010). Obstetric outcome in grand multiparous women in Jos teaching hospital. Jos J Med.

[14] Toohey JS, Keegan KA, Morgan MA, Francis J, Task S, deVeciana M (1995). The “dangerous multipara”: fact or fiction?. Am J Obstet Gynecol.

[15] Alsammani M, Ahmed S. Grand multiparity: risk factors and outcome in a tertiary hospital: a comparative study. Mater Sociomed. 2010;26(3):181–97 Available from: https://www.ncbi.nlm.nih.gov/pmc/articles/PMC4610637/. 10.5455/msm.2015.27.244-247 PMC4610637 26543415

[16] Ministry of Health Community Development Gender Elderly and Children. The National Road Map Strategic Plan to Improve Reproductive, Maternal, Newborn, Child and Adolescent Health in Tanzania (2016–2020) (One Plan II). 2016. Available from: https://www.globalfinancingfacility.org/sites/gff_new/files/Tanzania_One_Plan_II.pdf. Accessed 15 Dec 2018.

[17] Idoko P, Nkeng G, Anyawu M (2016). Reasons for current pregnancy amongst grand multiparous Gambian women - a cross sectional survey. BMC Pregnancy Childbirth.

[18] Afolabi AF, Adeyemi AS (2002). Extreme grand multiparity: is it an obstetric risk factor?. Eur J Obstet Gynecol Reprod Biol.

[19] Minkoff H, Grunebaum AN, Schwarz RH, Feldman J, Cummings M, Crombleholme W (1984). Risk factors for prematurity and premature rupture of membranes: a prospective study of the vaginal flora in pregnancy. Am J Obstet Gynecol.

[20] Ananth CV, Oyelese Y, Srinivas N, Yeo L, Vintzileos AM (2004). Preterm premature rupture of membranes, intrauterine infection, and oligohydramnios: risk factors for placental abruption. Obstet Gynecol.

[21] Okonofua FE (1997). Gynecology and obstetrics risk factors for primary postpartum haemorrhage. Arch Gynecol Obstet.

[22] Combs CA, Murphy EL, Laros RK (1991). Factors associated with postpartum hemorrhage with vaginal birth. Obstet Gynaecol.

[23] Drife J (1997). Management of primary postpartum haemorrhage. BJOG An Int J Obstet Gynaecol..

